# Assessing the Accuracy of Contrast-Enhanced CT Abdomen in Identifying and Stratifying Risk of Oesophageal Varices in Cirrhotic Patients: A Retrospective Study

**DOI:** 10.7759/cureus.94163

**Published:** 2025-10-08

**Authors:** Hayley J Collins, Shivakumar Chitturi, Shashank Sharma, Tarun Jain

**Affiliations:** 1 Department of Radiology, Canberra Hospital, Canberra, AUS; 2 Gastroenterology and Hepatology Unit, Canberra Hospital, Canberra, AUS

**Keywords:** cirrhosis, computed tomography, gastric varices, oesophageal varices, oesophagoduodenoscopy

## Abstract

Purpose: Oesophagogastroduodenoscopy (OGD) is the current standard for diagnosing and grading oesophageal varices (OV), but it is invasive and costly. This study retrospectively evaluated how well contrast-enhanced computed tomography (CT) scans of the abdomen perform in detecting and assessing the risk level of OV compared to OGD.

Methods: The study analyzed 92 cases of patients with liver cirrhosis who underwent contrast-enhanced CT within three months of having an OGD. Two experienced radiologists measured the largest OV diameter on CT images, which was then compared to OV grading from the OGD. Patient demographics, underlying causes of cirrhosis, and liver function classification were also reviewed.

Results: Detection rates of varices by the two radiologists were moderate, with a combined sensitivity under 70%. For mild varices, CT showed moderate sensitivity and specificity but low detection rates. For moderate and severe varices, CT sensitivity and specificity varied, with overall accuracy just over 50%. There was no clear correlation between varix size on CT and variceal grade on OGD.

Conclusion: Contrast-enhanced CT has limited accuracy in detecting and grading OV compared to OGD, highlighting that OGD remains the preferred method for OV evaluation in patients with cirrhosis.

## Introduction

Approximately 50% of patients newly diagnosed with cirrhosis are found to have gastrointestinal varices. Portal hypertension results in the abnormally dilated submucosal veins throughout the gastrointestinal tract. These varices pose a serious risk due to their potential to cause life-threatening bleeding. Oesophageal varices (OV) develop in approximately 5% of patients with cirrhosis by the end of the first year and increase to around 28% by the end of three years. Once present, small varices enlarge at an annual rate of approximately 10-12%. The yearly risk of bleeding is estimated at 5% for small varices and rises to 15% for large varices [1,2]. In cases of initial (index) variceal bleeding, the six-week mortality rate is estimated to be between 15% and 25% [1-3]. Without endoscopic treatment, the risk of rebleeding is nearly 60%, with a corresponding increase in mortality [1,3].

Determining the presence and size of varices and the presence of associated red wale marks requires oesophagogastroduodenoscopy (OGD), which is considered to be the “gold standard” [3]. It is recommended to screen all patients who are newly diagnosed with cirrhosis for OV [1]. OGD is also performed for surveillance of OV in cirrhotic patients. In cases of compensated cirrhosis, OGD is repeated at an interval of 1-3 years, depending on whether varices were present initially or not and whether ongoing liver injury and other risk factors are present [1,3,4]. In addition, patients with no or small varices on screening endoscopy should have a repeat endoscopy performed when and if decompensation develops [3].

OGD is an invasive and expensive procedure that can be associated with complications [1,3]. The Baveno VII consensus states that noninvasive tests are sufficiently accurate to identify clinically significant portal hypertension in clinical practice. However, many of their recommendations revolved around liver stiffness measurements by elastography, which is not yet widely available [4]. Various noninvasive methods have been studied to predict the presence of varices in patients with liver cirrhosis. These include serum-based markers such as the aspartate aminotransferase-to-platelet ratio index (APRI), aspartate aminotransferase-to-alanine aminotransferase ratio (AAR), and the fibrosis index based on 4 factors (FIB-4) scores, as well as measurements of liver and spleen stiffness, and the platelet count-to-spleen diameter ratio [5]. Despite these efforts, the overall diagnostic accuracy of these tools has been limited, which has restricted their routine use in clinical settings [5]. Spleen stiffness measurement with elastography is also included in the Baveno VII guidelines [4]. However, the best criteria to apply in clinical practice are still a matter of debate [6-9]. 

A computed tomography (CT) scan is a noninvasive, widely available technique that is extensively used to evaluate patients with liver cirrhosis. In addition, it can directly visualise the OV, most commonly identified at the distal oesophagus and the oesophagogastric junction [10-14].

This retrospective study involved the review of 92 cases (71 patients) with liver cirrhosis who underwent both contrast-enhanced abdominal CT and OGD. CT images were independently assessed by two radiologists, and their findings were compared against OGD results, which served as the reference standard.

The primary objective was to determine the diagnostic accuracy of CT in detecting the presence of OV. The secondary objective was to evaluate the ability of CT to identify high-risk varices, potentially impacting clinical decision-making. Through this analysis, the study aimed to explore whether CT can serve as a reliable, noninvasive alternative to OGD for variceal assessment in cirrhotic patients.

## Materials and methods

This retrospective study was approved by the institutional review board. Informed consent was not required for the review of medical records because a waiver was granted, allowing for the review of medical records without patient authorization.

Sample

Medical records were reviewed to identify patients diagnosed with liver cirrhosis who underwent OGD at The Canberra Hospital between January 2019 and April 2021. A total of 154 cases were included in which patients with cirrhosis also had a contrast-enhanced CT scan performed within three months of the OGD. The imaging for patients was performed at our institution as well as external imaging providers. 

CT scans were performed using standard imaging protocols, including axial and multiplanar reconstructions. Only studies with adequate portal venous phase imaging, as confirmed by the reporting radiologist, were included in the analysis. Multiphase studies were included if the portal venous phase met the inclusion criteria.

**Figure 1:**
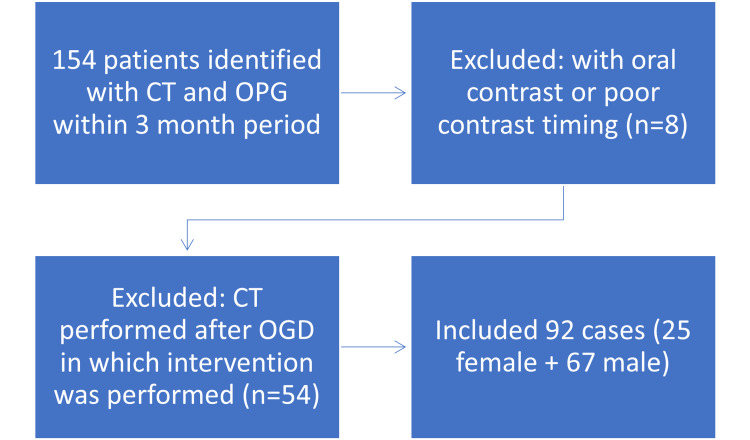
Flow chart of patient exclusion criteria CT: computed tomography; OGD: oesophagogastroduodenoscopy

Patients were excluded if the CT scan had suboptimal contrast timing, making accurate variceal measurement unreliable (n = 8), or if CT was performed after an OGD involving therapeutic intervention, such as band ligation (n = 54). The final study population consisted of 92 cases (71 patients) (Figure 1). For these cases, electronic medical records were reviewed for demographic details, blood test results, and OGD findings. Underlying causes of cirrhosis included hepatitis B and C, alcohol use, nonalcoholic steatohepatitis (NASH), and other etiologies. Three patients were ultimately excluded as they did not fulfill the diagnostic criteria for cirrhosis. The OGD report served as the reference standard for each case. Endoscopic findings were obtained retrospectively through review of the documented procedure notes. Elastography was not routinely utilized. Patients were considered eligible for OGD screening based on the Baveno VII criteria if they had a platelet count below 150 × 10⁹/L. Endoscopic grading of OV was categorized using the Pacquet classification: grade I (varices barely elevated above the mucosa), grade II (occupying one-third of the oesophageal lumen and noncompressible), and grade III (occupying up to 50% of the lumen). For this study, high-risk varices were defined as those with increased wall tension on endoscopy; these included grade II or higher, the presence of red wale markings, recent bleeding stigmata, or those requiring endoscopic therapy [1].

In this retrospective study, multiple OGD-CT episodes were included for individual patients to reflect real-world clinical practice. All clinically and temporally distinct episodes were included for a patient were included when justified by a new indication, evolving clinical status, or adequate time since the previous procedure. Each episode was treated as a separate event for both data collection and statistical analysis, provided it met the inclusion criteria.

CT

Image Analysis

Two board-certified radiologists with 17 (TJ) and five (SS) years of experience in interpreting CT abdomen images independently reviewed all images. Both observers were blinded to patient physical findings, laboratory data, earlier imaging findings, and endoscopic results.

**Figure 2:**
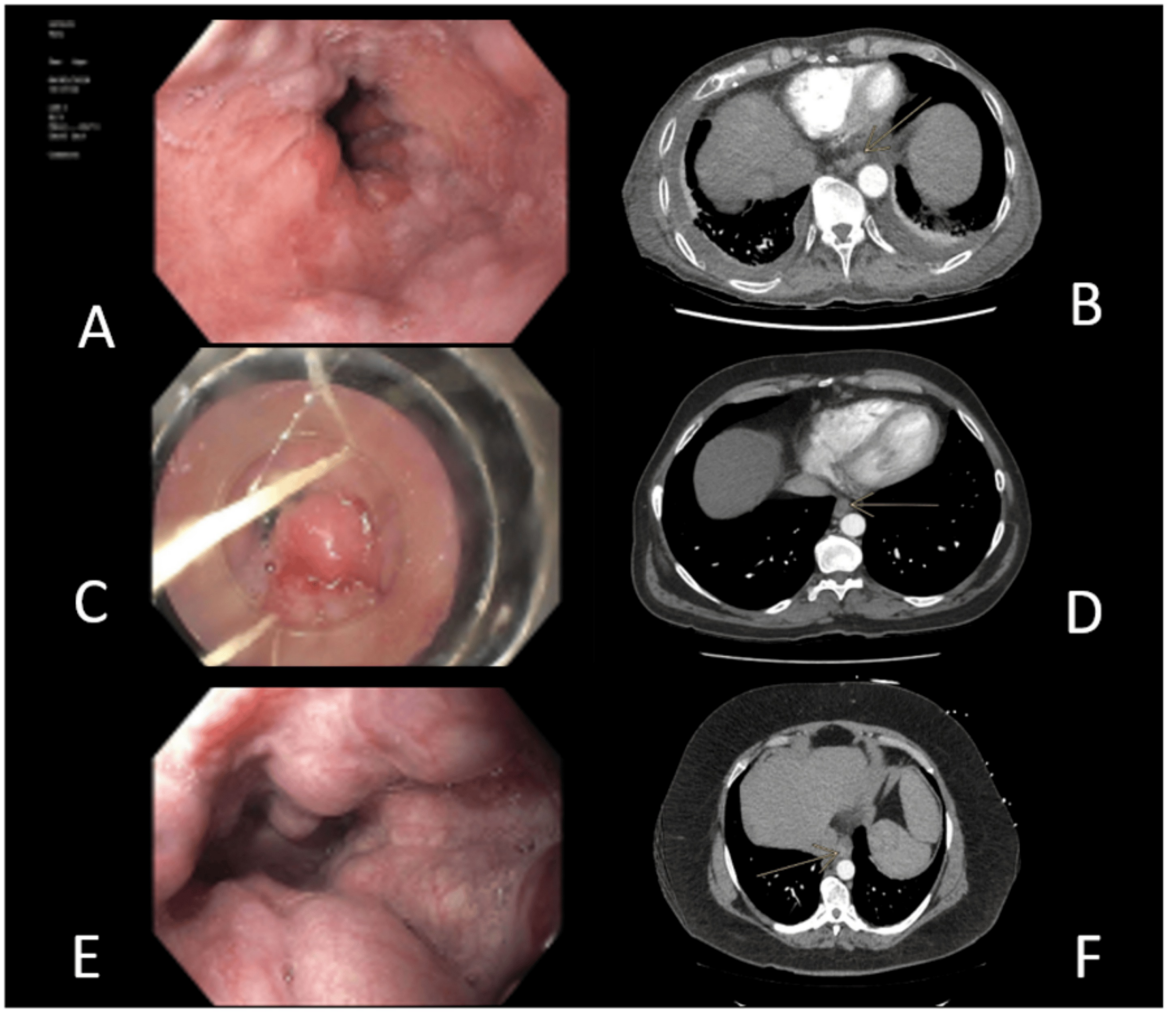
Oesophageal varices located in the lower 1/3 of oesophagus OGD: oesophagogastroduodenoscopy; CT: computed tomography; OV: oesophageal varices OGD (A) and corresponding arterial phase contrast-enhanced CT (B) of a patient with grade I OV  OGD (C) and corresponding arterial phase contrast enhanced CT (D) of a patient with grade II OV OGD (E) and arterial phase contrast enhanced CT (F) of a patient with grade III OV

**Figure 3:**
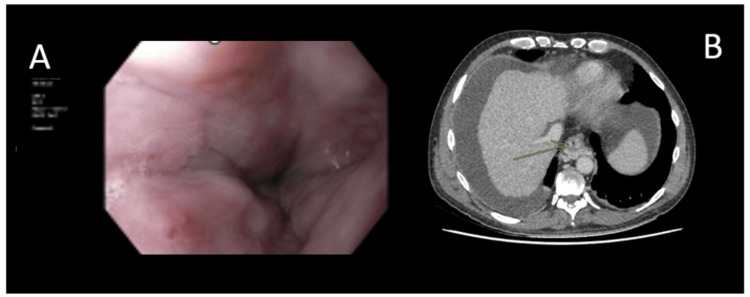
OGD (A) and corresponding portal phase contrast enhanced CT (B) of a patient with a red whale sign OGD: oesophagogastroduodenoscopy; CT: computed tomography

**Figure 4:**
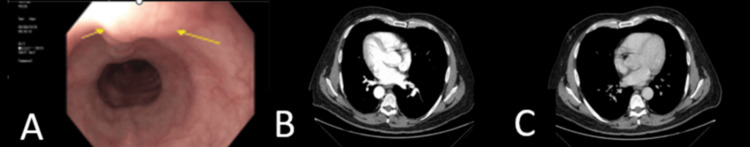
OGD (A) and corresponding arterial phase contrast-enhanced CT (B) and portal venous phase contrast-enhanced CT (C) of a patient with a false-negative CT interpretation OGD: oesophagogastroduodenoscopy; CT: computed tomography

Enhancing vessel in the oesophageal wall or protruding into the lumen was accepted as a positive finding for the presence of OV (Figure 2). The CT images in the portal venous or arterial phase were evaluated for the presence and size of the largest OV. Dependent on the tortuosity of the varix, the imaging plane that best enabled measurement of the shortest perpendicular diameter was selected. Measurements were recorded in millimetres to one decimal place (Figures 2-4). All measurements were performed on the portal venous phase images for consistency. 

Paraesophageal varices are defined as varices external to the muscular oesophageal wall without mass effect on the oesophageal lumen. As paraoesophageal varices would not be visible or require intervention during OGD and are not at high risk of rupture, they were not measured.

Data Analysis

**Figure 5:**
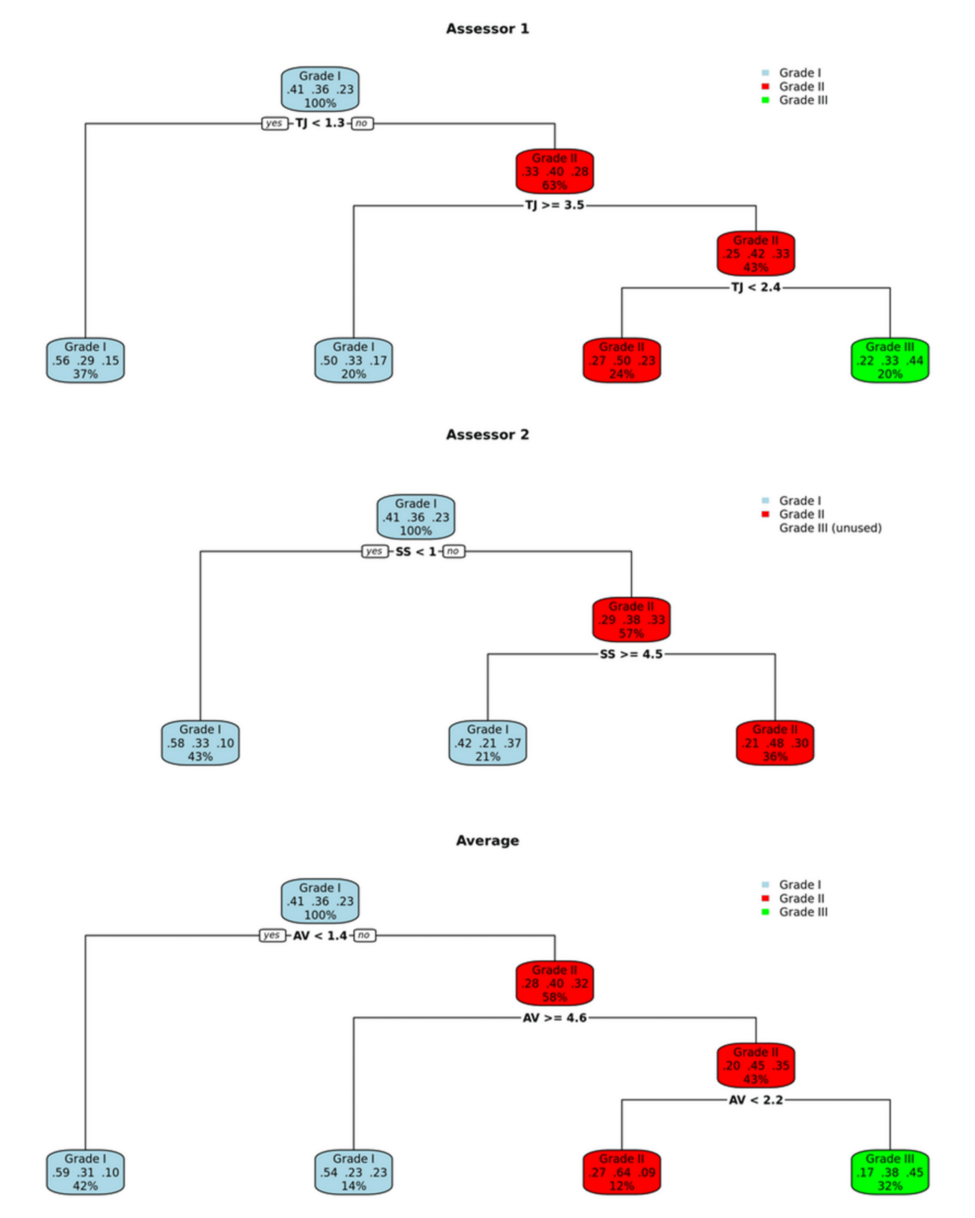
Decision trees generated to classify OV grades. Average decision tree was created with the average of the measures from the two readers OV: oesophageal varices

Continuous variables were summarized using means and standard deviations, while categorical variables were presented as absolute counts and percentages. To assess interobserver variability between the two radiologists, each assessor independently measured the imaging parameters used in the study. A decision tree method was then applied separately to the measurements from each assessor, as well as to the average of both assessors’ measurements, to determine optimal cut-off values. Decision trees were chosen because they clearly identify the most predictive variables and provide an easily interpretable classification process. Additionally, they handle complex interactions between variables without requiring strict statistical assumptions, making them well-suited for clinical decision-making. For each decision tree classification, sensitivity, specificity, positive predictive value (PPV), and negative predictive value (NPV) were calculated. Cohen’s kappa coefficients were used to quantify agreement both between each decision tree classification and the reference OGD classification, and between the two assessors’ classifications themselves. This approach allowed evaluation not only of the overall diagnostic performance of the decision trees but also of how interobserver variability influenced classification agreement and reliability (Figure 5). All analyses were performed using R [15], version 4.3.3, and the “rpart” package [16].

## Results

Patient characteristics

A total of 92 OGD procedures were performed in 71 patients, with a CT scan available within three months of the OGD. There were 67 males and 25 females with a mean age of 58.3 years. According to the Child-Pugh classification, 46 patients were classified as class A, 26 patients as class B, and 15 patients as class C. Patient demographics, causes of cirrhosis, clinical and laboratory characteristics, and Child-Pugh classes are given in Table 1.

**Table 1:** Baseline demographic data NASH: nonalcoholic steatohepatitis Multiple endoscopic procedures have been included for a single patient. The percentages listed here are of the 92 procedures rather than the 71 patients. (A) Other causes include autoimmune hepatitis, primary sclerosing cholangitis, and metastatic disease. (B) Other causes include stent removal or undocumented. (C) Patients had multiple causes of cirrhosis documented, all of which are included in this table

		Patient group	
Characteristic	All (n = 92)	Low-risk varices (n = 47)	High-risk varices (n = 45)
Sex, no (%)			
Male	67 (72.8)	33 (70.2)	34 (75.56)
Female	25 (27.2)	14 (29.79)	11 (24.44)
Cause of cirrhosis (%)^C^			
Hepatitis C or B	32 (34.8)	1 (2.13)	31 (68.89)
Alcohol	32 (34.8)	24 (51.06)	8 (17.78)
NASH	9 (9.8)	3 (6.38)	6 (13.33)
Noncirrhotic	3 (3.2)	3 (6.38)	0 (0)
Other^A^	16 (17.4)	16 (34.04)	0 (0)
Child-Pugh class (%)			
A	49 (53.3)	29 (61.7)	20 (44.44)
B	27 (29.3)	18 (38.30)	9 (20.00)
C	15 (16.3)	7 (14.89)	8 (17.78)
Unclassified	1 (1.0)	1 (1.5)	0 (0.0)
Indication for endoscopy			
Surveillance	50 (54.3)	32 (40.4)	31 (68.89)
Bleeding	23 (25.0)	15 (31.91)	8 (17.78)
Other^B^	19 (20.4)	13 (27.66)	6 (13.33)
Prior procedures (%)			
No	36 (39.1)	24 (51.06)	12 (26.67)
Yes	32 (34.8)	12 (25.53)	20 (44.44)
Missing	24 (26.1)	11 (23.40)	13 (28.89)

Endoscopic findings

Of the 92 cases included, varices were identified on OGD in all patients (100%). High-risk varices, based on endoscopic findings, were present in 45 patients (48.9%). A total of 37 patients (40.2%) had grade I OV, 33 (35.9%) had grade II OV, and 22 (23.9%) had grade III OV. Red wale signs or stigmata of recent bleeding were documented in seven patients (7.7%).

These features were identified retrospectively through review of the original OGD reports. The presence of red wale signs or bleeding stigmata was only recorded if explicitly noted by the endoscopist in the report. While reporting standards may vary slightly between endoscopists, the documentation of such high-risk features is routine clinical practice at our centre and was found to be consistent across reports included in the study. Low- and high-risk varices were observed across all Child-Pugh classes. No other significant oesophageal structural abnormalities, such as masses, were reported.

Diagnostic performance of CT

The decision tree method has found different classification rules for the two readers (Figure 5). The accuracy of both readers was 51.1% (95% CI: 40.4%-61.7%) when predicting the grade of the varices. For grade I OV, CT exhibited a sensitivity of 78.9%, specificity of 59.3%, PPV of 57.7%, and a detection rate of 32.6%. For grade II OV, CT exhibited a sensitivity of 21.2%, specificity of 93.2%, and PPV of 63.6%. For grade III OV, CT exhibited a sensitivity of 61.9%, specificity of 77.5%, and PPV of 44.8%.

On the other hand, the variceal detection rates for the readers were 67.0% (reader 1) and 54.9% (reader 2), with a combined detection rate of 69.2%, when at least one reader detected OV. The overall accuracy of CT in detecting OV was 96.7% (95% CI: 90.7%-99.3%), 84.6% (95% CI: 75.5%-91.3%), and 98.9% (95% CI: 94.0%-99.9%), for readers 1, 2, and the combined detection. The kappa values observed were 92.3%, 67.7%, and 97.4%, respectively (Figure 5). The increasing size of OV on CT did not correlate with the increasing grade of OV on OGD (Table 2).

**Table 2:** Statistics by OGD grade OGD: oesophagogastroduodenoscopy; PPV: positive predictive value; NPV: negative predictive value

	Grade I	Grade II	Grade III
Sensitivity	0.7368	0.3333	0.38095
Specificity	0.5556	0.8136	0.85915
PPV	0.5385	0.5	0.44444
NPV	0.75	0.6857	0.82432

## Discussion

In this study, we aimed to analyse the accuracy of a standard portal venous phase CT abdomen in identifying OV. OV meet several key criteria to require screening and surveillance, including a clearly defined at-risk population (patients with cirrhosis), the significant cost, morbidity, and mortality linked to associated complications (such as haemorrhage), and the existence of effective preventive therapies, such as β-blockers or endoscopic band ligation [4].

Complications of cirrhosis are often evaluated with a CT abdomen. The additional evaluation of OV on these scans provides an opportunity for interpreting radiologists to add value to the report [8]. The overall acceptance of patients for CT has been previously evaluated to be significantly more than that for endoscopy [10].

Previous studies have evaluated the use of CT as a screening modality for OVs. In two meta-analyses, the sensitivity of CT scan for the detection of OV was found to be 0.87-0.91, and the specificity was 0.75-0.8 [4,9]. A study by Manchec et al., which found a size of at least 4 mm, was 80% sensitive (95% CI, 67-89%) and 87% specific for high-grade OV [8]. Studies that specifically examined the optimal variceal size on CT for achieving the highest sensitivity and specificity identified a threshold of 3-4 mm as most effective [8].

This compares with our study, in which the overall accuracy of CT for the detection of OV was calculated at 85-99%. While the NPV for grade III OV of 87.30% could be used to suggest that patients without CT measurable OV are unlikely to have grade III OV, this was less helpful for grade II OV (67.90%) and grade I OV (80.00%).

Our study has some important limitations that must be considered. First, CT examinations were performed across multiple institutions with variations in scanning protocols, which may have introduced heterogeneity affecting diagnostic consistency. Although scans with suboptimal portal venous phase enhancement were excluded, the lack of a standardized protocol limits generalizability and could have influenced accuracy.

Previous studies have used specialized protocols to enhance variceal assessment, including CT oesophagography with oesophageal air insufflation via intubation [11], triple-phase CT combined with effervescent granules [13], intravenous administration of butyl scopolamine (Buscopan, Sanofi Australia) to induce hypotonia [10], and thin-slice image reconstructions [12]. These techniques improve diagnostic precision but are cumbersome and not practical in routine clinical practice.

While OGD remains the gold standard for evaluating OV, its inherent subjectivity complicates direct comparison. Significant interobserver and intraobserver variability in endoscopic grading has been documented, though this study did not assess these inconsistencies [12].

Additionally, the interval between OGD and CT of up to three months may have allowed changes in variceal size to occur, introducing temporal bias. Previous studies have used shorter intervals to minimize this effect [8,10]. The study also did not assess patients’ medication profiles, such as the use of portal pressure-reducing drugs, which may affect variceal size and CT detectability.

Finally, the retrospective design and nonconsecutive case selection limit the applicability of results to a screening population. Despite this, the proportion of high-risk varices in our cohort was similar to prospective studies, suggesting that PPVs remain relevant [14]. 

## Conclusions

This study demonstrates that contrast-enhanced portal venous phase CT shows variable performance in detecting OV across different grades. For grade I varices, CT exhibited moderate sensitivity (78.9%) but relatively low specificity (59.3%) and PPV (57.7%), reflecting limitations in accurately identifying early-stage varices. The sensitivity for grade II varices was notably low at 21.2%, despite high specificity (93.2%) and a PPV of 63.6%, indicating a risk of underdetection at this stage. For grade III varices, CT showed improved sensitivity (61.9%) and specificity (77.5%), though the PPV remained moderate at 44.8%.

Interobserver variability was evident, with variceal detection rates ranging from 54.9% to 67.0% between readers, and a combined detection rate of 69.2% when at least one reader identified varices. Overall diagnostic accuracy was high, reaching up to 98.9% with combined detection. The agreement between readers, quantified by kappa values, varied from moderate (67.7%) to excellent (97.4%), underscoring variability in image interpretation.

These findings indicate that while CT demonstrates high overall accuracy and can be a valuable adjunctive tool, its limited sensitivity, particularly for grade II varices, and variability between readers suggest that it cannot replace OGD as the gold standard. The statistical measures highlight the need for cautious interpretation of CT results and support its role as a complementary rather than standalone modality in variceal assessment.
